# Exercise-Mobilized Donor Lymphocytes Enhance Blinatumomab Activity Against B-ALL

**DOI:** 10.21203/rs.3.rs-10395685/v1

**Published:** 2026-08-13

**Authors:** John L Sullivan, Geovana Leite, Nina Santa Cruz, Helena Batatinha, Kaitlin A Swanson, Dimitrios Filioglou, Grace M McKenzie, Timothy M Kistner, Prabhu S Arunachalam, Forrest L Baker, Emmanuel Katsanis, Richard Simpson

**Affiliations:** The University of Arizona; The University of Arizona; The University of Arizona; The University of Arizona; The University of Arizona; The University of Arizona; The University of Arizona; The University of Arizona; The University of Arizona; University of South Carolina; The University of Arizona; The University of Arizona College of Medicine – Tucson: The University of Arizona College of Medicine Tucson

**Keywords:** T-cell engager, Adoptive cell therapy, Graft-versus-leukemia, Exercise immunology, T-cell receptor signaling, NKG7, Cytotoxic lymphocytes, CD137, 4–1BB, C134, OX40

## Abstract

**Background:**

Donor lymphocyte infusion (DLI) is used to prevent and treat leukemic relapse after allogeneic hematopoietic cell transplantation, although its efficacy against lymphoid malignancies remains limited. Blinatumomab (Blina), a CD3/CD19 bispecific T-cell engager, is increasingly used to enhance DLI activity against acute lymphoblastic leukemia (ALL). We previously demonstrated that exercise-mobilized DLI (DLI-X) exhibits superior anti-leukemic activity against myeloid malignancies. Here, we investigated whether DLI-X combined with Blina enhances cytotoxicity against CD19^+^ B-ALL.

**Methods:**

Peripheral blood mononuclear cells (PBMCs) were collected from healthy participants at rest (DLI) and during the final stage of a 20-minute graded cycling protocol to 80% VO_2_max (DLI-X). Cytotoxicity against CD19^+^ RS4;11 B-ALL cells was assessed in vitro. T-cell activation and effector function were evaluated by 8-color flow cytometry. In vivo efficacy was determined in NSG-IL-15 mice engrafted with RS4;11 cells and treated with DLI or DLI-X in combination with Blina or vehicle, with tumor burden monitored by bioluminescence imaging.

**Results:**

DLI and DLI-X alone exhibited limited cytotoxicity against RS4;11 cells. Blina significantly enhanced cytotoxicity of both products, with DLI-X+Blina demonstrating >20% greater tumor killing than DLI+Blina (~85% vs. ~62%). Compared with DLI, DLI-X displayed enhanced Blina-induced T-cell effector function, including increased CD107a degranulation, greater TNF-α production by CD4^+^ T cells, higher IFN-γ production by CD8^+^ T cells, and 10–15% greater upregulation of CD137 and OX40. In vivo, DLI-X+Blina achieved up to 35% greater control of tumor burden than DLI+Blina.

**Conclusions:**

Exercise mobilizes T cells with enhanced responsiveness to Blina, resulting in superior activation and cytotoxicity against CD19^+^ B-ALL cells in vitro and improved tumor control in vivo. These findings support the use of exercise-mobilized DLI as a strategy to enhance the efficacy of bispecific T-cell engager therapy following allogeneic hematopoietic cell transplantation.

**Trial registration:**

ClinicalTrials.gov, NCT06643221. Registered retrospectively on October 8, 2024.

## Introduction

Allogeneic hematopoietic cell transplantation (allo-HCT) remains a potentially curative therapy for patients with high-risk or relapsed hematologic malignancies. However, post-transplant relapse continues to be a major cause of treatment failure. Donor lymphocyte infusion (DLI) is commonly given after allo-HCT to augment the graft-versus-leukemia (GvL) effect by supplying additional donor-derived T-cells capable of recognizing and eliminating residual malignant cells.^1^ While highly effective in chronic myeloid leukemia (CML),^2^ DLI has shown more limited success in acute leukemias, where long-term survival remains modest (~ 25% two-year OS)^3^. These limitations are particularly evident in B-cell acute lymphoblastic leukemia (B-ALL), a disease in which relapse after allo-HSCT remains common and therapeutic options are limited, especially in adult patients who experience substantially worse outcomes than pediatric populations^4,5,6^.

The treatment landscape for relapsed or refractory (R/R) B-ALL has evolved significantly with the introduction of targeted immunotherapies, including the CD19 × CD3 bispecific T-cell engager (BiTE^®^) Blina.^7,8^ Blina simultaneously binds CD19 on leukemic cells and CD3 on T-cells, promoting immunological synapse formation and T-cell activation. This interaction triggers serial killing of CD19-positive leukemic cells through perforin- and granzyme-mediated cytotoxicity, independently of MHC Class I recognition.^9,7^ In the post-transplant setting, Blina is increasingly used in combination with DLI to enhance donor T-cell–mediated GvL activity^10,11,12,13,1^. However, efficacy depends on the composition and functional competence of cytotoxic lymphocytes within the DLI product, which vary substantially between donors and often lack adequate frequencies of highly cytotoxic effector subsets.^2,3,4^

Physical activity is emerging as a modifiable behavioral intervention with potent immunomodulatory effects that may enhance the efficacy of T-cell-based immunotherapies, including Blina and DLI.^14^ Acute bouts of dynamic exercise induce a rapid mobilization of immune cells into the circulation, primarily driven by catecholamine signaling through β_2_-adrenergic receptors on lymphocytes, which reduces endothelial adhesion and promotes their release into the bloodstream^15,16,17,18^. This response preferentially mobilizes highly differentiated cytotoxic populations, including natural killer (NK) cells and effector CD8^+^ T-cells, generating a transiently enriched pool of immune cells with phenotypic and transcriptional profiles associated with enhanced cytotoxicity, tissue migration, and immune surveillance. Recent preclinical studies suggest that adoptive transfer of exercise-mobilized lymphocytes from a healthy donor (referred to as DLI-X) may enhance GvL activity. In xenogeneic mouse models, DLI-X improved anti-tumor responses without exacerbating graft-versus-host disease (GvHD)^18^, and when combined with exercise-mobilized cytokine-activated NK cells further enhanced cytotoxic activity against myeloid leukemia cells and improved leukemia control *in vivo*^19^. Together, these findings suggest that acute exercise may provide a practical strategy to enrich DLI products with highly cytotoxic immune subsets^14^; however, whether exercise-mobilized donor lymphocytes can enhance T-cell–dependent immunotherapies such as the CD3-engaging bispecific antibody Blina remains unknown.

To address this gap, we investigated whether DLI-X could enhance Blina-mediated anti-leukemic responses comparing the functional activity of standard DLI and exercise-mobilized donor lymphocytes *in vitro* and *in vivo*. DLI-X demonstrated greater synergy with Blina than standard DLI, with enhanced leukemia cell cytotoxicity, increased T-cell degranulation and cytokine secretion, enrichment of activated phenotypes (e.g., CD137^+^ and OX40^+^ T-cells), and improved control of human B-ALL in xenogeneic mouse models. These findings indicate that acute exercise can be leveraged as a simple and biologically grounded strategy to generate an optimized donor lymphocyte product for T-cell–mediated immunotherapy after allo-HCT.

## Methods

### Donor Participants

Twenty-nine healthy adults (Table 1) participated in this study, recruited on our registered clinical trial ‘Exercise as an Immune Adjuvant for Allogeneic Cell Therapies (Allo-X)’ (ClinicalTrials.gov ID NCT06643221). Written informed consent and medical history were obtained prior to each visit. Participants arrived after an overnight fast and abstained from vigorous physical activity for 24 hours beforehand. All participants were considered ‘low risk’ for maximal graded exercise testing per AHA/ACSM guidelines.^20^ The study was approved by the Human Subjects Protection Program at the University of Arizona and conducted in accordance with the Declaration of Helsinki (#1801161041).

### Exercise Trials and Blood Sampling

Participants completed two laboratory visits separated by 1–2 weeks. During visit 1, participants were screened and completed a maximal graded cycling protocol (Lode Excalibur Sport) with continuous heart rate and gas exchange monitoring (Cosmed CPET, Rome, Italy), beginning at 50W with 15W increments per minute until volitional exhaustion or failure to maintain > 60 RPM. VO_2_max and peak power were used to assign individualized workloads for visit 2. During visit 2, resting venous blood was collected into EDTA vacutainers (Becton-Dickinson, USA), followed by a 20-minute graded cycling protocol at 50%, 60%, 70%, and 80% VO_2_max (5 minutes each). Blood was collected during the final 1–3 minutes of the 80% stage. A 1h post-exercise sample was collected to observe egress only and was not used in functional assays.

### Blood Processing and Analysis

Whole blood was analyzed for complete blood counts (Beckman Coulter, Indianapolis, IN) and lymphocyte phenotyping by flow cytometry (MACSQuant 16, Miltenyi Biotec, San Diego, CA) using fluorescent monoclonal antibodies (Miltenyi anti-CD8, CD3, CD4, CD56, CD14, CD16, CD20, CD62L, CD45RA, TCR-γδ, PD-1, and KLRG1). PBMCs were gated by forward/side scatter in FlowLogic (Inivai Technologies, Australia), with single-color tubes for compensation and FMOs for gating validation; a minimum of 100,000 PBMCs were acquired per sample. Resting PBMCs (DLI) and exercise-mobilized PBMCs (DLI-X) were used fresh for in vivo studies or cryopreserved at 10×10^6^ cells/mL (90% FBS, 10% DMSO) for in vitro analysis.

### Surface and Intracellular Phenotyping

PBMCs were labeled with Miltenyi or BD fluorescent monoclonal antibodies. T-cells were defined as CD45^+^/CD3^+^/CD4^+^ or CD8^+^, with subsets defined by CD62L and CD45RA: naïve (CD62L^+^/CD45RA^+^), CM (CD62L^+^/CD45RA^−^), EM (CD62L^−^/CD45RA^−^), and EMRA (CD62L^−^/CD45RA^+^). Unconventional T-cells were CD3^+^/CD4^−^/CD8^−^; NK and NKT cells were delineated by CD56 and CD3 expression. Intracellular NKG7 staining used the Miltenyi Inside Stain Kit (130-090-477) with unconjugated anti-NKG7 (Thermofisher) and Goat anti-Rabbit FITC secondary antibody. Additional Miltenyi antibodies included anti-CD45, TCR-γδ, CD20, CD56, CD62L, CD45RA, NKG2A, NKG2C, NKG2D, TRAIL, FasL, CD137, OX40, KLRG1, CTLA-4, and CRTAM.

### T-cell Cytolysis Assays

RS4;11-Luc (B-ALL) and Jeko-1 (mantle cell lymphoma) cells were thawed and cultured 72 hours prior to use. RS4;11-Luc cells were seeded at 0.3×10^6^ cells/mL in RPMI with 10% FBS at 37°C, 5% CO_2_. Target cells (20,000/well) were plated in 96-well plates with Blina (66 ng/mL) and co-cultured with PBMCs or negatively selected CD4^+^ or CD8^+^ T-cells (Miltenyi kits 130-096-533 and 130-096-495; >90% purity) at E:T ratios of 0–20:1, alongside a serial dilution standard curve (40,000–2,500 cells). After 24 hours at 37°C, 5% CO_2_, 10 μL D-Luciferin potassium salt (15 mg/mL in DPBS; Gold Biotechnologies, St. Louis MO) was added per well and plates were imaged at 10-second exposure on the LargoX bioluminescence imager. Luminescence was quantified in photons/s using Aura Imaging software. Percent cytolysis was calculated as: Live cells = m(photons/s) + b; %Cytolysis = 100 − (Live cells/(20,000 × 100)).

### Assessment of T-cell Degranulation and Cytokine Secretion

PBMCs were co-cultured with RS4;11 or Jeko-1 at a 1:1 E:T ratio overnight at 37°C, 5% CO_2_ in the presence of anti-human CD107a antibody ± Blina (66 ng/mL). Brefeldin A (GolgiPlug; BD Biosciences) and Monensin (GolgiStop; BD Biosciences) were added for 2.5 hours to block protein transport. After surface staining for CD3, CD4, and CD8, cells were fixed, permeabilized, and stained intracellularly for IFN-γ and TNF-α. Samples were analyzed on a BD LSR Fortessa^®^ flow cytometer with single-color and FMO controls.

### Animal Experiments

Female NSG-IL15 mice (NOD.Cg-Prkdcscid Il2rgtm1Wjl Tg(IL15)1Sz/SzJ; 8–12 weeks; The Jackson Laboratory) were bred in-house under specific-pathogen-free conditions with a 12-hour light/dark cycle. On day − 1, mice were irradiated (150 cGy; RadSource X-ray irradiator) and on day 0 injected intravenously with 500,000 RS4;11-luc cells in PBS. On day + 10, DLI or DLI-X (3×10^6^ T-cells/mouse)^2^^1^ was administered intravenously, and Blina (62.5 ng in 200 μL PBS) or vehicle was given intraperitoneally on days + 10–17. Body weight was recorded twice weekly; mice with > 10% sustained weight loss or hind-limb paralysis were euthanized. Tumor burden was monitored by BLI (200 μL D-luciferin, 15 mg/mL; GoldBio Cat#Luck-1g; 2% isoflurane anesthesia) using the Lago-X system (Spectral Instruments, Tucson, AZ) with Aura imaging software at a 5-minute exposure.

### Statistical Analysis

Data are presented as mean ± SD. In vivo BLI data were analyzed in R (v4.5.1) using a linear mixed-effects model with log-transformed tumor burden as the outcome, including fixed effects for treatment group, time, and their interaction, with random intercepts at the donor level and for mice nested within donors. A Vehicle + Blina group was omitted as prior experiments showed no Blina effect without DLI. DLI+Blina versus DLI-X+Blina comparisons used estimated marginal means. Survival was analyzed by Kaplan-Meier method and Cox proportional hazards models with donor-level random effects. In vitro analyses used GraphPad Prism v10.6.1 (paired t-tests, mixed-effects models, or repeated-measures ANOVA as detailed in figure captions). Statistical significance was set at p < 0.05.

## Results

### Exercise preferentially mobilizes cytotoxic EM and EMRA CD8 + T-cells expressing NKG7

Acute exercise induced an approximately 3.7-fold increase in circulating monocytes and a 2-fold increase in circulating lymphocytes (Table 2). All major lymphocyte subsets were mobilized in response to exercise, with the largest relative increases observed for NK cells and EMRA CD8 + and CD4 + T-cell subsets. After 1 h of recovery, circulating monocyte counts and all major lymphocyte subsets had returned to baseline or decreased below pre-exercise values (Table 2). To determine whether acute exercise alters the expression of receptors associated with cytotoxic effector function in circulating T-cells, peripheral blood mononuclear cells were analyzed by flow cytometry at rest and during exercise at 80% VO_2_max. Among the markers examined (NKG2D, NKG2A, NKG2C, and NKG7), only NKG7 expression differed significantly between resting and exercise-mobilized T-cells (Fig. 1a,b). NKG7 is an intra-vesicular protein associated with exocytosis and release of cytolytic granules, primarily in NK cells,^22^ however, studies have identified NKG7 as a key player in degranulation among cytotoxic CD8 + T-cells,^23^ which could be imperative in Blina-mediated T-cell effector function. Exercise increased the frequency of NKG7^+^ CD8^+^ T-cells while reducing NKG7 expression among CD4^+^ T-cells (Fig. 1a,b).

T-cell subsets were defined by CD45RA and CD62L expression to identify naïve, central memory (CM), effector memory (EM), and EMRA populations. Acute exercise reduced the proportion of naïve cells within both CD4^+^ and CD8^+^ compartments and increased the proportion of CD8^+^ EMRA cells, while other subsets did not change significantly (Fig. 1c,d). Consistent with this redistribution, NKG7 expression was constitutively highest among CD8^+^ EM and EMRA T-cells. However, NKG7 expression also increased within CD8^+^ CM T-cells despite no change in their relative abundance (Fig. 1e), indicating that the exercise-induced increase in NKG7^+^ CD8^+^ T-cells reflects both preferential mobilization of subsets with constitutively higher cytotoxic protein expression and increased NKG7 expression within specific populations, potentially due to mobilization of CD8^+^ T-cells with a cytotoxic phenotype that retain a CM surface profile. Naïve subsets were suppressed in exercise mobilized T-cells, which are typically implicated in GvHD.^24,25,26^ Together, these findings demonstrate that acute exercise mobilizes circulating T-cells enriched for cytotoxic-associated proteins, supporting the concept that exercise-mobilized lymphocytes may provide a functionally enhanced donor lymphocyte product.

### Exercise-mobilized lymphocytes exhibit enhanced Blina-dependent cytotoxicity in vitro

To determine whether exercise-mobilized lymphocytes exhibit enhanced cytotoxicity against lymphoid tumors in the presence of Blina, we performed 24-hour *in vitro* cytotoxicity assays using luciferase-expressing RS4;11 cells (Fig. 2). In the absence of Blina, cytotoxicity at 24 hours was low and comparable between resting and exercise-mobilized PBMCs across a range of E:T ratios. However, in the presence of Blina, exercise-mobilized PBMCs demonstrated significantly greater tumor cell killing than resting PBMCs against RS4;11 cells (Fig. 2a). To determine whether T-cells accounted for this enhanced cytotoxicity, CD4^+^ and CD8^+^ T-cells were purified from resting and exercise-mobilized PBMCs and co-cultured with RS4;11 target cells. Consistent with whole PBMC assays, exercise-mobilized T-cells exhibited greater cytotoxicity than resting T-cells, but only in the presence of Blina. Both CD4^+^ and CD8^+^ T-cells mediated effective tumor cell killing under these conditions (Fig. 2b,c). Together, these findings demonstrate that exercise-mobilized lymphocytes exhibit enhanced Blina-dependent cytotoxic activity against CD19^+^ lymphoid tumor cells.

### Blina enhances degranulation and cytokine production in exercise-mobilized T-cells

To determine whether enhanced Blina-dependent cytotoxicity involved increased T-cell effector activity, we assessed degranulation and cytokine production following 24-hour co-culture of RS4;11 or Jeko-1 target cells with resting or exercise-mobilized PBMCs at a 1:1 E:T ratio with and without Blina. In the absence of Blina, resting and exercise-mobilized T-cells displayed comparable levels of degranulation and cytokine production. As expected, Blina markedly increased T-cell effector responses. Under these conditions, exercise-mobilized T-cells exhibited significantly greater degranulation than resting T-cells, as indicated by CD107a expression, TNF-α and IFN-γ (Fig. 3a,b). The strongest response of IFN-γ production was observed in CD8^+^ T-cells, followed by CD4^+^, while exercise-mobilized CD4^+^ T-cells displayed increased TNF-α expression compared with resting T-cells (Fig. 3c–f). Together, these findings indicate that exercise mobilization enhances Blina-induced T-cell effector responses.

### Blina-induced costimulatory receptor expression is enhanced in exercise-mobilized T-cells

To further characterize the mechanisms underlying enhanced Blina-dependent cytotoxicity, we measured surface expression of activating and costimulatory receptors on T-cells after stimulation with Blina. PBMCs obtained at rest and during exercise were co-cultured with Jeko-1 cells at a 1:1 ratio and analyzed at 0, 24, 48, and 72 hours for expression of CD137 (4–1BB), OX40 (CD134), and FasL. CRTAM, TRAIL, NKG2A, NKG2C and NKG2D expression were also analyzed but showed no upregulation with Blina. In the absence of Blina, expression of these markers remained low and did not differ between resting and exercise-mobilized lymphocytes. Blina stimulation significantly increased expression of CD137, OX40, and FasL over time in both CD4^+^ and CD8^+^ T-cells (Fig. 4a,b,c).

Among these markers, CD137 expression was higher but not significant in exercise-mobilized CD4^+^ and CD8^+^ T-cells compared with resting T-cells following Blina stimulation after 24 hours (Fig. 4b). OX40 expression was similarly increased in exercise-mobilized CD4^+^ T-cells but not CD8^+^ T-cells (Fig. 4c)

To further characterize the responding population, CD137^+^ T-cells were analyzed 24 hours after activation (Fig. 5a). Exercise mobilization skewed the CD137^+^ population toward a CD8-dominant phenotype (Fig. 5b) with increased expression of the differentiation marker KLRG1, but not OX40, FasL, NKG2D, PD-1, or CTLA-4 (Fig. 5d). This shift corresponded with enrichment of effector memory (EM) and EMRA subsets among CD137^+^ CD8^+^ T-cells (Fig. 5c). Among CD4^+^ T-cells, exercise modestly increased the representation of EM and central memory subsets within the CD137^+^ population, accompanied by a slight increase in KLRG1 expression (Fig. 5e). In addition, exercise increased the proportion of NK cells within the overall CD137^+^ lymphocyte population. Together, these data indicate that exercise mobilization enhances Blina-induced activation of cytotoxic lymphocyte populations, particularly CD8^+^ T-cells with effector phenotypes.

### Exercise-mobilized lymphocytes enhance Blina-mediated graft-versus-leukemia activity in vivo

To determine whether exercise-mobilized lymphocytes enhance Blina-dependent tumor control *in vivo*, NSG-IL15 mice were sublethally irradiated and injected intravenously with 5 × 10^5^ luciferase-expressing RS4;11 cells. After a 10-day engraftment period, mice received donor lymphocyte collected either at rest (DLI) or during exercise (DLI-X) normalized to 3 × 10^6^ T-cells per animal. Mice were then treated with daily intraperitoneal Blina or vehicle from days 10–17, and tumor burden was assessed by bioluminescence imaging (Fig. 6a).

DLI-X products were enriched for cytotoxic lymphocyte populations, with higher frequencies of CD8 + T-cells, unconventional T-cells (defined as CD3+/CD4−/CD8−), NK, NKT (defined as CD56+/CD3+), and NK Dim cells, and lower frequencies of CD4 + T-cells compared with DLI when normalized per 100 T-cells. Frequencies of double positive T-cells, γδ T-cells, B-cells, and NK bright cells were unchanged (Fig. 6b).

Mice receiving DLI-X plus Blina showed the greatest tumor control, with the lowest bioluminescence signal across the treatment period. DLI or DLI-X alone did not significantly alter tumor progression compared with vehicle. Both DLI and DLI-X reduced tumor burden in combination with Blina, with a greater effect observed in the DLI-X + Blina group (Fig. 6c). Maximal differences in tumor burden were observed around day 17, near the end of treatment. After cessation of Blina treatment, tumor burden returned in both DLI and DLI-X groups but remained lower in mice receiving DLI-X.

Donor cell engraftment was similar between DLI and DLI-X groups, with comparable levels of total T-cells and T-cell subsets. However, in the presence of Blina, both CD4^+^ and CD8^+^ T-cells expanded to a greater extent in mice receiving DLI-X compared with DLI (Fig. 6f). GvHD morbidity scores did not differ between groups. (Fig. 6e) Overall survival was reduced in tumor-bearing mice receiving vehicle compared with those treated with DLI or DLI-X, whereas no survival differences were observed among groups receiving DLI or DLI-X with or without Blina (Fig. 6f).

Together, these data demonstrate that exercise-mobilized lymphocytes enhance Blina-mediated GvL activity *in vivo* without increasing GvHD.

## Discussion

Allo-HCT remains the only curative option for many patients with high-risk B-ALL. However, post-transplant relapse is common and many patients require DLI to maintain GvL activity. Despite this, DLI is frequently insufficient, highlighting the need for strategies that enhance the efficacy of donor-derived immune responses^27,28,29,30,31,1^. Recent work from our group has shown that exercise-mobilized lymphocytes may represent an improved source of DLI products, in part due to their enrichment for NK cells and enhanced activity against myeloid leukemias^18,14,32,33^. However, it remained unclear whether this advantage would extend to lymphoid malignancies, where anti-tumor responses depend more heavily on T-cell function and, increasingly, on combination strategies such as bispecific antibody-mediated T-cell redirection.

In this study, we identify a novel approach to augment DLI efficacy against lymphoid leukemia using exercise-mobilized lymphocytes (DLI-X) in combination with the CD19 × CD3 bispecific T-cell engager Blina. While Blina is increasingly used in relapsed/refractory B-ALL and in combination withDLI,^34,35,36,12,37,38,10^ responses remain variable and are likely dependent on the composition and functional competence of donor T-cells. Our findings demonstrate that acute exercise can be leveraged to improve the functional quality of DLI products and enhance their responsiveness to T-cell–redirecting immunotherapy.

A key observation from this study is that acute exercise mobilizes a population of T-cells with enhanced cytotoxic potential at both the phenotypic and functional level. We show that acute exercise preferentially redistributes CD8^+^ effector memory (EM) and EMRA T-cells, consistent with prior work demonstrating selective mobilization of highly differentiated cytotoxic subsets^39,40,18,15,41,42,43^. In parallel, exercise increased expression of NKG7 within CD8^+^ T-cells, a protein associated with cytolytic granule exocytosis and degranulation^23,44^. Notably, this increase was observed not only through redistribution of EM/EMRA populations with constitutively high NKG7 expression, but also within CD8^+^ central memory (CM) T-cells, suggesting that exercise mobilizes a subset of cells with cytotoxic capacity that is not fully captured by conventional surface phenotyping. EM and EMRA subsets drive GvL, whereas naive and CM cells promote GvHD. Because the latter are relatively depleted in DLI-X, enhanced GvL without concurrent GvHD, decoupling of the two, would be the predicted outcome.^24,25,26^ These findings support the concept that exercise mobilization enriches for lymphocytes with enhanced intrinsic cytolytic machinery, which may be particularly relevant for therapies such as Blina that rely on rapid serial killing^45,46^.

Consistent with this phenotype, exercise-mobilized lymphocytes exhibited enhanced Blina-dependent cytotoxicity *in vitro*. Importantly, this effect was not observed in the absence of Blina, indicating that exercise does not simply increase baseline T-cell cytotoxicity but instead enhances the ability of T-cells to respond to CD3-engaging bispecific activation. Both CD4^+^ and CD8^+^ T-cells independently exhibited enhanced cytotoxic activity following exercise mobilization, suggesting that exercise broadly enhances the effector potential of multiple T-cell subsets. These findings are further supported by increased degranulation and cytokine production following Blina stimulation, with exercise-mobilized CD8^+^ T-cells exhibiting greater CD107a expression and IFN-γ production, and CD4^+^ T-cells producing more TNF-α. Together, these data indicate that exercise mobilization enhances multiple dimensions of T-cell effector function in the context of bispecific antibody engagement.

Mechanistically, we observed enhanced induction of costimulatory pathways following Blina activation in exercise-mobilized lymphocytes. Expression of CD137 (4–1BB) was significantly increased in both CD4^+^ and CD8^+^ T-cells, while OX40 expression was selectively enhanced in CD4^+^ T-cells. These receptors are critical regulators of T-cell expansion, survival, and effector function and have been implicated in the efficacy of multiple T-cell–based immunotherapies.^47,48^ Further characterization of the CD137^+^ population revealed a shift toward a CD8-dominant, KLRG1^+^ effector phenotype, with enrichment of EM and EMRA subsets and increased representation of NK cells. This suggests that exercise mobilization not only enhances activation but also shapes the composition of the responding population toward highly differentiated cytotoxic effectors. Given that CD137^+^ T-cells have been proposed as a functionally enriched subset for adoptive cell therapy^49,50,51^. these findings raise the possibility that exercise may provide a non-invasive strategy to generate DLI products enriched for highly responsive effector populations.

Importantly, these mechanistic and *in vitro* findings translated into improved anti-leukemic activity *in vivo*. In a xenogeneic CD19 + lymphoid leukemia model, DLI-X combined with Blina produced the most effective tumor control, whereas DLI or DLI-X alone had minimal impact. These results highlight the dependence of DLI efficacy on T-cell redirection and suggest that exercise-mobilized lymphocytes are particularly well suited to cooperate with bispecific antibody therapies. Notably, enhanced tumor control in DLI-X was accompanied by greater expansion of both CD4^+^ and CD8^+^ T-cells *in vivo* in the presence of Blina, consistent with our *in vitro* observations of increased activation and costimulation.

Among these improvements in GvL activity, we did not observe differences in GvHD severity between treatment groups. GvHD remains a major limitation of DLI, driven primarily by alloreactive T-cells. While we hypothesized that DLI-X would mitigate GvHD because of its augmented lymphocyte population, this effect was not recapitulated in the current lymphoid model. This may reflect fundamental differences in disease biology, as lymphoid malignancies are more directly targeted by T-cells, which are also the principal mediators of GvHD. These findings suggest that while DLI-X enhances anti-leukemic activity, it does not improve or worsen GvHD. Additional strategies may be required to dissociate GvL from GvHD in this setting, like GvHD mitigating combination therapies.^33^ However, given that the composition of DLI-X is shifted to a less GvHD prone phenotype, the expected outcome would be that DLI-X eould suppress GvHD.^24,25,26^

Several limitations should be considered. First, the *in vivo* administration of Blina in this study does not fully replicate the continuous infusion used clinically, which may influence pharmacokinetics and treatment efficacy. However, recent reports have shown efficacy with Blina given subcutaneously.^52^ Second, while our data demonstrate consistent enhancement of T-cell function following exercise mobilization, the degree of this effect may vary between donors, which could influence the reproducibility of DLI-X in a clinical setting. Finally, although our findings support a mechanistic role for enhanced costimulatory activation, additional studies will be required to fully define the molecular pathways underlying these effects.

In conclusion, acute exercise mobilizes a population of human lymphocytes enriched for cytotoxic effector function and enhanced responsiveness to CD3-mediated activation. Consequently, exercise-mobilized lymphocytes demonstrate increased Blina-dependent cytotoxicity *in vitro* and improved GvL activity *in vivo*, associated with enhanced effector function and costimulatory receptor expression. These findings support the use of exercise-mobilized donor lymphocytes as a simple and biologically grounded strategy to enhance the efficacy of Blina and other T-cell–redirecting therapies in patients with relapsed or refractory B-ALL.

## Figures and Tables

**Figure 1:**
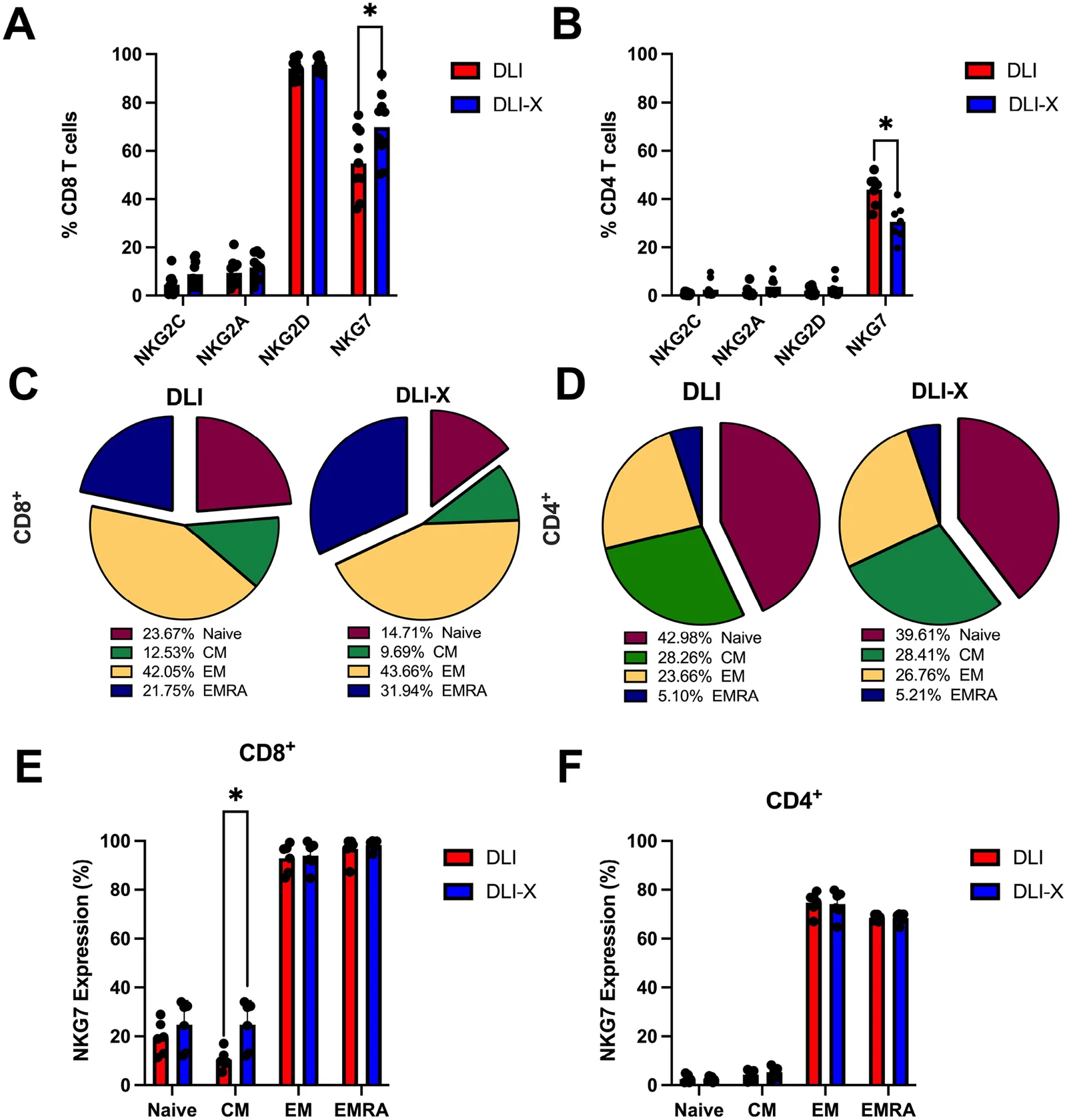
Exercise preferentially mobilizes cytotoxic EM and EMRA CD8^+^ T cells expressing NKG7. PBMCs were collected from 6 healthy donors at rest (DLI) and during the final stage of a graded cycling exercise protocol at ~80% VO_2_max (DLI-X) and analyzed by multicolor flow cytometry. (A) Frequency of CD8^+^ T cells expressing NKG2C, NKG2A, NKG2D, and NKG7 at rest (red) and during exercise (blue). (B) Frequency of CD4^+^ T cells expressing the same markers. (C, D) Proportional composition of CD8^+^ and CD4^+^ T cell subsets in DLI and DLI-X products, defined by co-expression of CD62L and CD45RA as naïve (CD62L^+^CD45RA^+^), central memory (CM; CD62L^+^CD45RA^−^), effector memory (EM; CD62L^−^CD45RA^−^), and terminally differentiated effector memory (EMRA; CD62L^−^CD45RA^+^). Separated pieces of the pie charts indicate statistically significant differences. *P ≤ 0.05 (E, F) NKG7 expression within each CD8^+^ and CD4^+^ T cell subset in DLI and DLI-X. Signoficance was determined by multiple paired t-tests. Significant differences between DLI and DLI-X indicated by *P ≤ 0.05.

**Figure 2:**
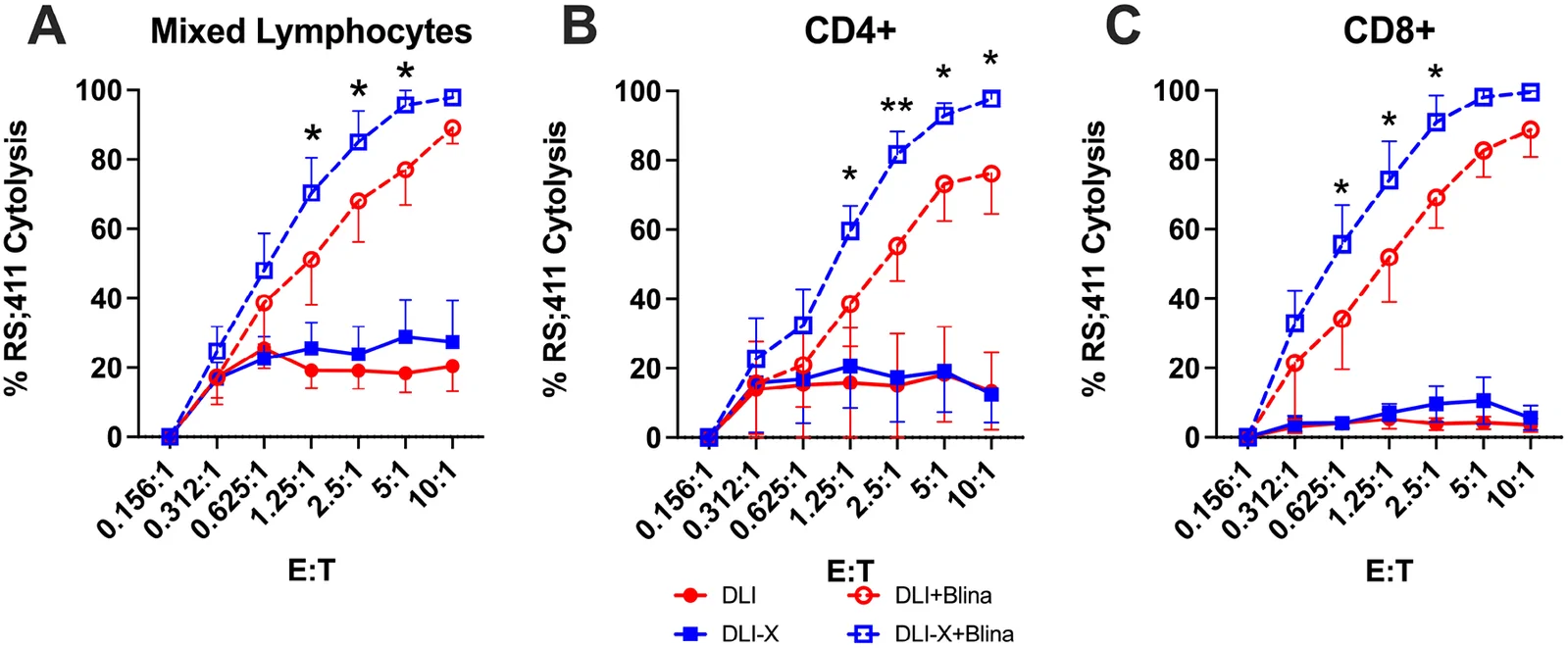
Exercise-mobilized lymphocytes exhibit enhanced blinatumomab-dependent cytotoxicity in vitro. PBMCs (N=10) or purified T cell subsets (N=5) from donors at rest (DLI) or during exercise (DLI-X) were co-cultured with luciferase-expressing RS4;11 cells across a range of effector-to-target (E:T) ratios in the presence or absence of blinatumomab for 24 hours at 37°C. Percent cytolysis was calculated by comparing bioluminescence to a standard curve of known target cell numbers and is displayed for DLI (red solid line), DLI-X (blue solid line), DLI + Blina (red dashed line), and DLI-X + Blina (blue dashed line). (A) Cytolysis by unseparated PBMCs (mixed lymphocytes). (B) Cytolysis by negatively selected CD4^+^ T cells. (C) Cytolysis by negatively selected CD8^+^ T cells. Significance was determined at each E:T ratio by 2way ANOVA. Asterisks denote significant differences between DLI + Blina and DLI-X + Blina at the indicated E:T ratio (*P ≤ 0.05, **P ≤ 0.01).

**Figure 3:**
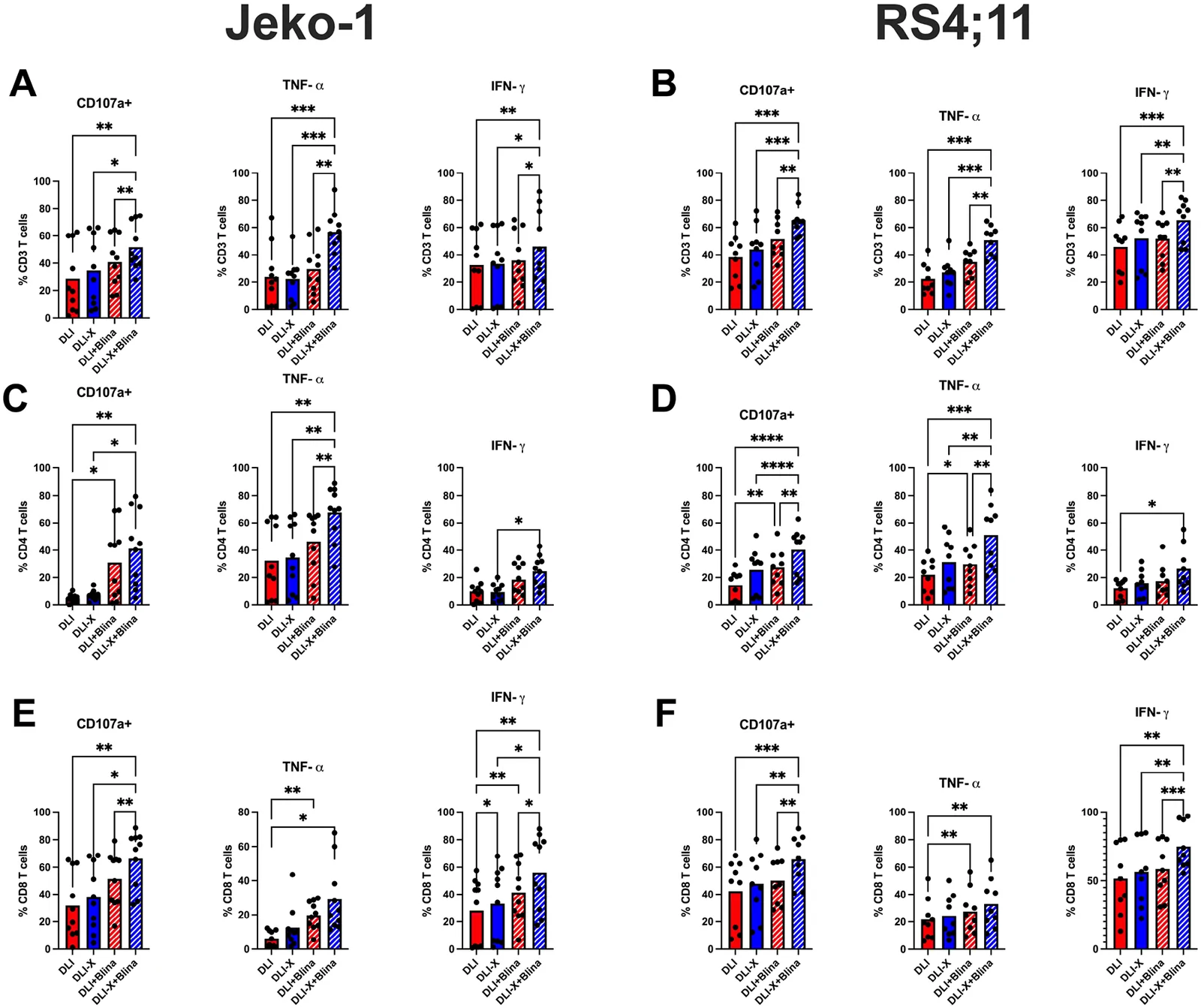
Blinatumomab enhances degranulation and cytokine production in exercise-mobilized T cells. PBMCs from 10 donors at rest (DLI) or during exercise (DLI-X) were co-cultured with RS4;11 (B-ALL) or Jeko-1 (mantle cell lymphoma) CD19^+^ target cells at a 1:1 effector-to-target ratio with or without blinatumomab for 24 hours at 37°C in the presence of anti-CD107a antibody. Following co-culture, cells were treated with Brefeldin A and Monensin for 2.5 hours prior to surface and intracellular staining for IFN-γ and TNF-α. Results are shown for DLI (red), DLI-X (blue), DLI + Blina (red striped), and DLI-X + Blina (blue striped). Data from Jeko-1 co-cultures are shown on the left half of the figure; RS4;11 co-cultures are shown on the right. (A,B) Degranulation (CD107a^+^), TNF-α, and IFN-γ expression among total CD3^+^ T cells against Jeko-1 or RS4;11 (C,D) The same markers measured within the CD4^+^ T cell compartment. (E,F) The same markers measured within the CD8^+^ T cell compartment. Significance was calculated by two-way ANOVA where *P ≤ 0.05, **P ≤ 0.01, ***P ≤ 0.001, ****P ≤ 0.0001.

**Figure 4:**
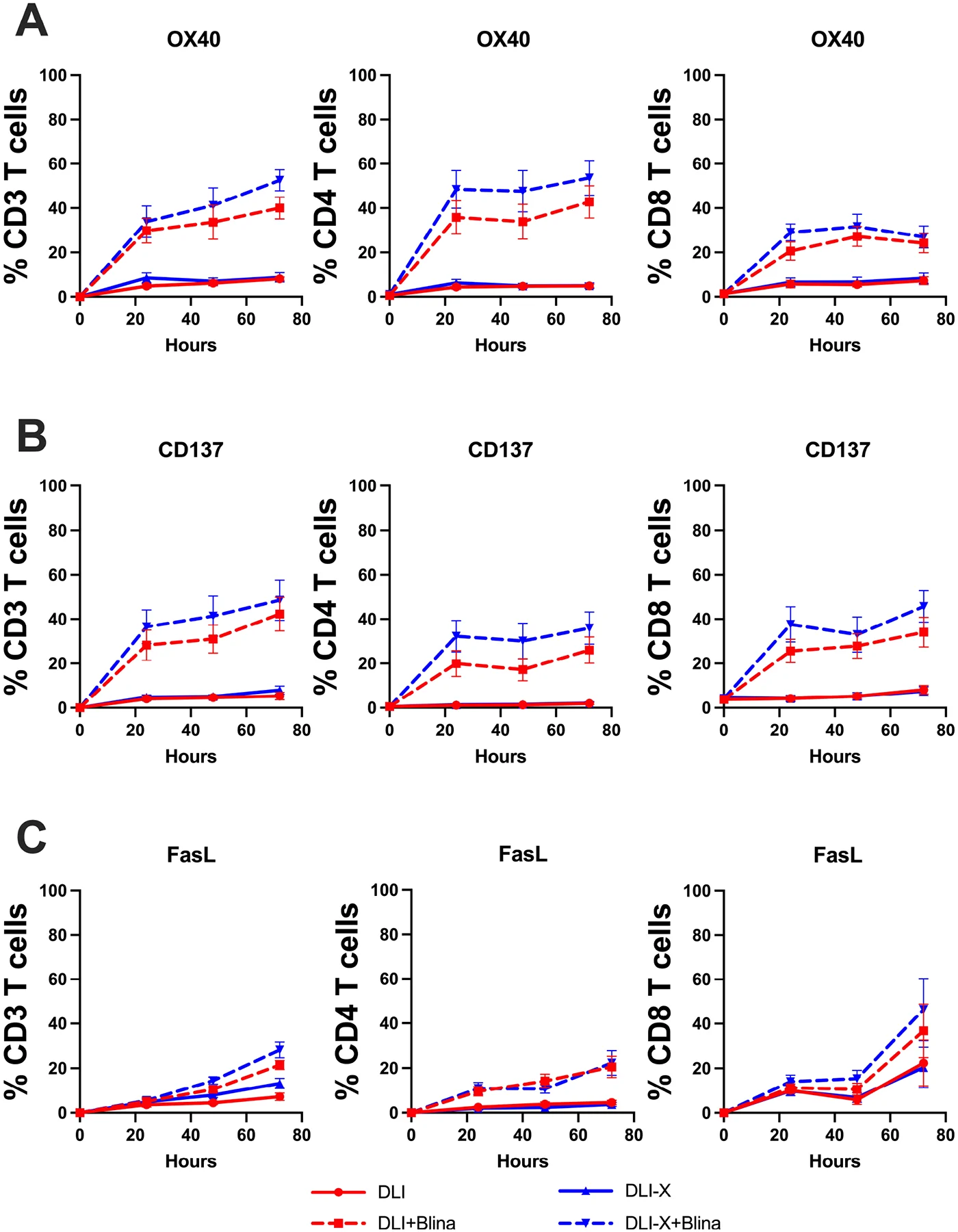
Blinatumomab-induced costimulatory receptor expression is enhanced in exercise-mobilized T cells. PBMCs from 10 donors at rest (DLI) or during exercise (DLI-X) were co-cultured with CD19^+^ Jeko-1 target cells at a 1:1 effector-to-target ratio with or without blinatumomab (66 ng/mL) at 37°C. Cells were collected at 0, 24, 48, and 72 hours and analyzed by flow cytometry for surface expression of OX40, CD137 (4–1BB), and FasL on T cell subsets. Data are shown for DLI (red solid line), DLI-X (blue solid line), DLI + Blina (red dashed line), and DLI-X + Blina (blue dashed line). (A) OX40 expression on total CD3^+^, CD4^+^, and CD8^+^ T cells over 72 hours. (B) CD137 expression on total CD3^+^, CD4^+^, and CD8^+^ T cells over 72 hours. (C) FasL expression on total CD3^+^, CD4^+^, and CD8^+^ T cells over 72 hours. Statistical analysis by two-way ANOVA did not show significant differences between groups.

**Figure 5:**
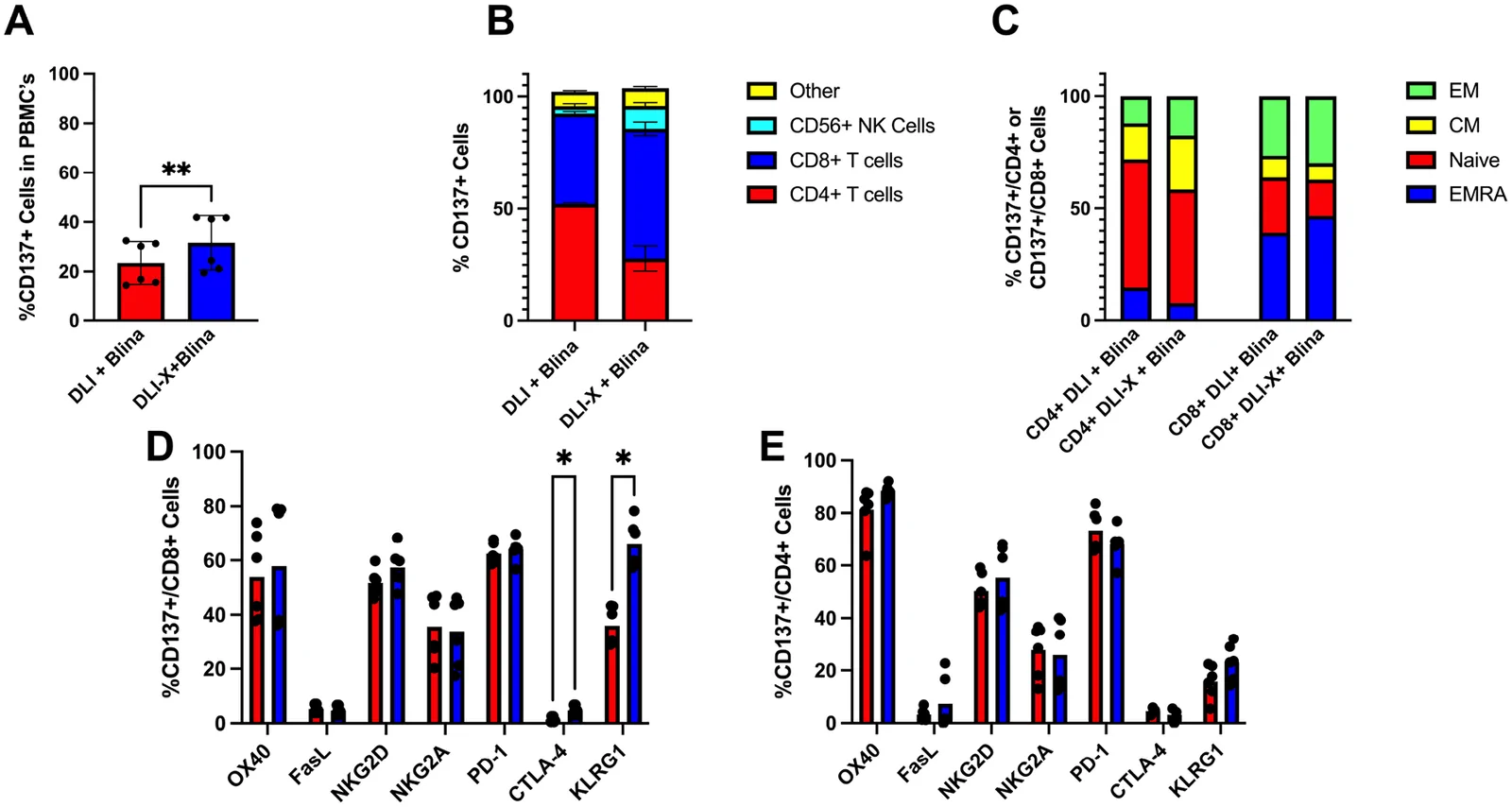
Exercise-mobilized T cells are enriched for effector-associated markers within the CD137^+^ population following blinatumomab stimulation. PBMCs from 6 donors at rest (DLI) or during exercise (DLI-X) were co-cultured with CD19^+^ Jeko-1 target cells at a 1:1 effector-to-target ratio with blinatumomab (66 ng/mL) for 24 hours at 37°C, then phenotyped by multicolor flow cytometry. (A) Frequency of CD137^+^ cells among total PBMCs in DLI (red) and DLI-X (blue) following stimulation. **P ≤ 0.01. (B) Cellular composition of the CD137^+^ population in DLI and DLI-X, showing proportions of CD4^+^ T cells (red), CD8^+^ T cells (blue), CD56^+^ NK cells (cyan), and other cells (yellow). (C) Memory subset composition of CD137^+^ CD4^+^ and CD137^+^ CD8^+^ T cells in DLI and DLI-X, defined by CD27 and CD45RA expression as naïve, CM, EM, and EMRA. (D) Co-expression of costimulatory, cytotoxic, and inhibitory surface markers — OX40, FasL, NKG2D, NKG2A, PD-1, CTLA-4, and KLRG1 — on CD137^+^ CD8^+^ T cells (left) and CD137^+^ CD4^+^ T cells (right) from DLI and DLI-X. *P ≤ 0.05. Statistical analysis was performed by multiple paired t-tests.

**Figure 6:**
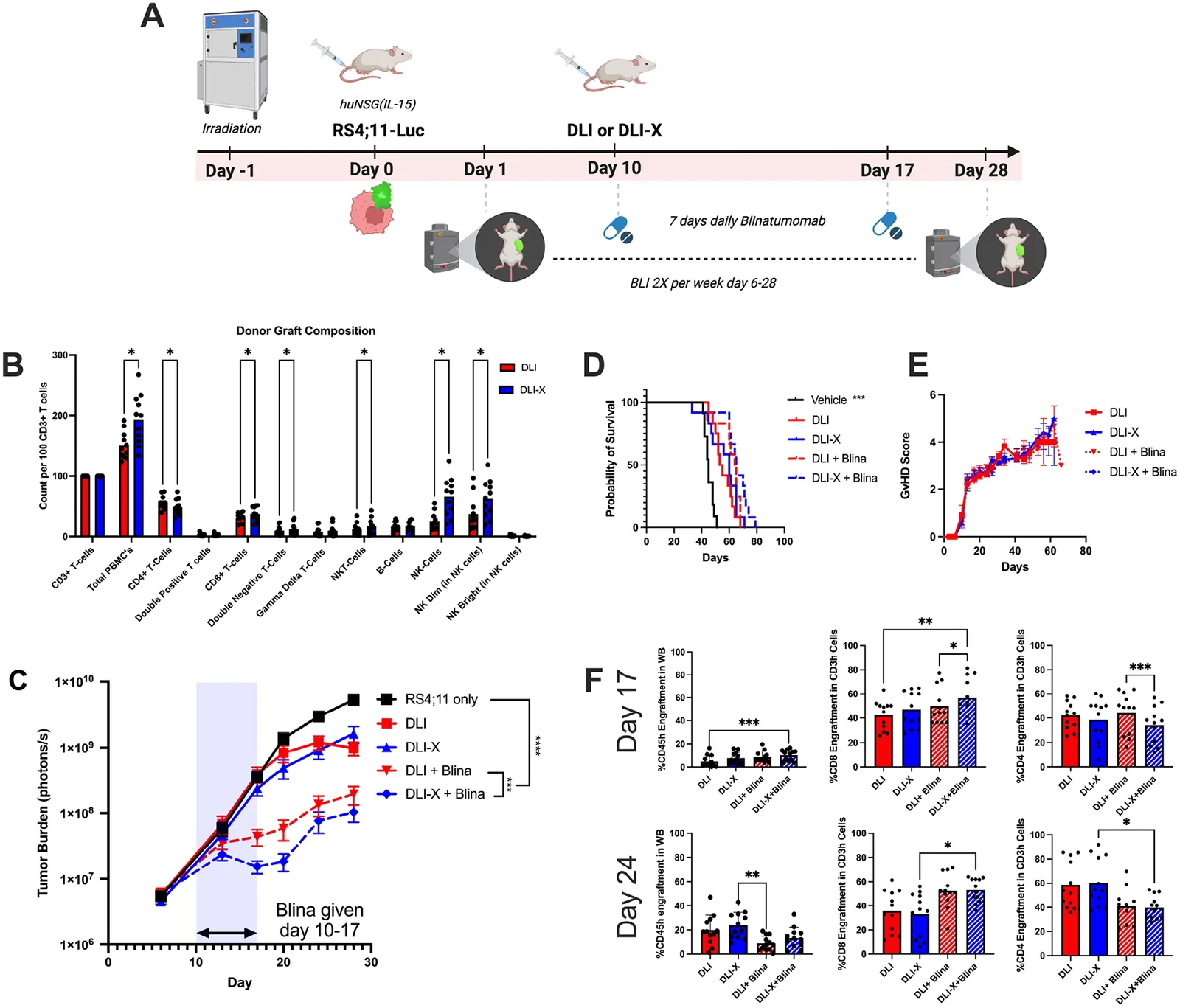
Exercise-mobilized lymphocytes enhance blinatumomab-mediated graft-versus-leukemia activity in hu-NSG-IL15 mice. (A) Experimental schematic. Female NSG-IL15 mice were sublethally irradiated (150 cGy) on day −1 and injected intravenously with 5 × 10^5^ luciferase-expressing RS4;11 cells on day 0. On day +10, mice received DLI or DLI-X normalized to 3 × 10^6^ T cells per mouse. Blinatumomab (62.5 ng) or vehicle was administered intraperitoneally daily from days +10–17. Bioluminescence imaging (BLI) was performed from days 6–28. Three independent experiments were conducted, each using a single healthy human donor, with 4 mice per group per experiment (n = 12 mice per group total). (B) Donor graft composition. PBMCs from each donor were analyzed by flow cytometry and cell counts were normalized per 100 CD3^+^ T cells to reflect the relative composition of DLI and DLI-X products across major lymphocyte subsets. *P ≤ 0.05 analyzed by multiple paired t-tests. (C) Tumor burden over time, quantified as total bioluminescence (photons/s) and displayed for RS4;11 only (black), DLI (red solid), DLI-X (blue solid), DLI + Blina (red dashed), and DLI-X + Blina (blue dashed). Shaded region indicates the blinatumomab treatment window (days 10–17). Significant differences between DLI + Blina and DLI-X + Blina, and between both blinatumomab-treated groups and vehicle control, are indicated. (D) Kaplan-Meier survival curves across treatment groups. All experimental groups receiving human effector cells were significantly different from vehicle control (***P ≤ 0.001); no survival differences were observed among DLI and DLI-X groups with or without blinatumomab. (E) GvHD clinical scores assessed twice weekly throughout the study. No significant differences in GvHD severity were observed between groups. (F) Human cell engraftment was assessed by tail bleed on day +17 (top row) and day +24 (bottom row) by flow cytometry. Total human CD45^+^ chimerism in whole blood (left), and frequencies of CD8^+^ (center) and CD4^+^ (right) T cells among CD3^+^ human cells are shown for each treatment group. *P ≤ 0.05, **P ≤ 0.01, ***P ≤ 0.001 by two way ANOVA

**Table 1:** Physical and demographic characteristics of the donor participants (n = 29)

ID	Sex	Age (years)	Weight (kg)	BMI	VO2max (mL/kg/min)	Peak power (W)	PA-R (1–7)	Fitness Score
**Donor_1**	F	34	74.5	26.4	33.7	200	7	Fair
**Donor_2**	M	40	91.6	29.9	35.8	260	5	Average
**Donor_3**	F	39	54.9	20.2	31.6	170	7	Fair
**Donor_4**	M	21	62.6	21.6	44.7	215	7	Very Good
**Donor_5**	M	25	82.5	24.6	41.5	275	6	Average
**Donor_6**	M	27	80	23.3	48.1	290	7	Very Good
**Donor_7**	M	27	80.4	23.6	49.9	295	7	Very Good
**Donor_8**	F	30	67.2	23.3	36.5	230	7	Average
**Donor_9**	M	30	112	29.5	20.1	200	5	Poor
**Donor_10**	F	30	52	21.6	48.8	230	7	Very Good
**Donor_11**	M	29	74	21.6	35.7	200	5	Average
**Donor_12**	F	27	57.3	23.2	35.9	170	6	Average
**Donor_13**	M	25	82	25.9	28.6	170	4	Fair
**Donor_14**	M	33	88.3	26.4	38.1	260	6	Average
**Donor_15**	F	26	67.8	24	32.8	200	4	Average
**Donor_16**	M	28	86.3	25.2	40.5	275	7	Average
**Donor_17**	M	21	70	23.4	58.3	305	7	Superior
**Donor_18**	M	26	86.6	24.8	40	260	5	Average
**Donor_19**	M	51	93.6	25.1	37.5	380	7	Average
**Donor_20**	M	27	95.6	27.0	41.5	335	7	Average
**Donor_21**	F	22	61	21.9	38.6	185	6	Poor
**Donor_22**	F	49	58.7	21.5	29.8	140	7	Fair
**Donor_23**	M	25	80.6	29.6	50.2	320	7	Very Good
**Donor_24**	F	27	56.3	21.3	43.6	185	6	Average
**Donor_25**	F	51	68.6	22.7	32.7	200	7	Very Good
**Donor_26**	F	30	49.5	17.6	35.2	125	7	Average
**Donor_27**	F	54	60.7	19.2	36.7	185	6	Very Good
**Donor_28**	F	40	53.2	20.8	39.14	185	6	Very Good
**Donor_29**	F	43	95.7	33.0	30.3	245	7	Very Good
**Average**	15M / 14 F	32.31	73.91	24.08	38.48	230.69	6.28	
**SD**		9.49	15.99	3.45	7.80	61.17	0.96	

PA-R questionnaire scoring(AS Jackson 1990): 0 to 1, do not participate regularly in physical activity; 2 to 3, participate regularly in light physical activity; 4 to 5, participate regularly in moderate physical activity; 6 to 7, participate regularly in heavy physical activity.

Cardiorespiratory fitness (VO_2_max) was age- and sex-adjusted and categorized according to normative values published by the American College of Sports Medicine ranging from “very poor” to “superior.”(M American College of Sports

ACSM’s Guidelines for Exercise Testing and Prescription

BMI, body mass index; Weight, body weight; mL, milliliters; kg, kilograms; min, minute; ID, identity; F, female; M, male; PA-R, physical activity rating; SD, standard deviation; W, Watts

**Table 2:** Exercise Preferentially Mobilizes Lymphocytes with Greater Effector Phenotypes (n = 29)

Leukocyte Subsets (cells/μL)	Rest	EX	+ 1HR	Time F (*p- value)*	Mobilization (Δ%)
**Lymphocytes**	1891 ± 560	3667 ± 996****	2055 ± 506*	32.8 (< *0.0001*)	94 ± 36
**CD3**^**+**^ **T-cells**	1184 ± 459	1764 ± 662****	1027 ± 403	44 (< *0.0001*)	49 ± 24
**CD4**^**+**^ **T-cells**	665 ± 274	857 ± 324****	569 ± 228	39.7 (< *0.0001*)	29 ± 21
**Naïve**	288 ± 146	363 ± 189***	222 ± 114	10 (*0.0009*)	26±18
**CM**	201 ± 106	261 ± 146***	171 ± 88	5.36 (*0.0003*)	30 ± 28
**EM**	153 ± 62	246 ± 94****	173 ± 70	10.81 (< *0.0001*)	60±17
**EMRA**	15 ± 62	48 ± 66*	21 ± 61	10.81 (*0.0223*)	212±60
**CD8**^**+**^ **T-cells**	396 ± 186	697 ± 336****	360 ± 184	15.59 (< *0.0001*)	76 ± 37
**Naïve**	92 ± 55	110 ± 70*	76 ± 56	2.43 (*0.0352*)	19±37
**CM**	51 ± 33	73± 55**	42 ± 28	3.725 (*0.0039*)	40 ± 41
**EM**	171 ± 109	327 ± 217***	167 ± 110	5.173 (*0.0004*)	90 ± 40
**EMRA**	63 ± 77	239 ± 258*	58 ± 100	2.9 (*0.0158*)	278 ± 71
**CD4**^−^ **CD8**^−^ **T-cells**	84 ± 86	158 ± 171****	61 ± 35	14.56 (< *0.0001*)	88 ± 51
**NK-cells**	364 ± 241	1298 ± 560****	248 ± 123**	51.03 (< *0.0001*)	257 ±165
**NKT-cells**	127 ± 112	246 ± 192****	96 ± 83*	35.32 (< *0.0001*)	93 ± 67
**B-cells**	204 ± 84	303 ± 126**	201 ± 78	4.8 *(0.0045)*	49 ± 35
**Monocytes**	331 ± 144	1233 ± 262****	218 ± 174*	53.21 (< *0.0001*)	273 ± 30

mL- microliter, CM – central memory, EM – Effector Memory, NK – Natural Killer cells, NKT – Natural Killer like NKT-cells

Data are mean ± standard deviation.

F, F-statistic.

Difference from rest indicated by *P ≤ .05, **P ≤ .01, ***P ≤ .001, ****P ≤ .0001, as determined using the Tukey multiple comparison test.

$$\text{Mobilization}\left(\%\Delta \right)=\frac{\text{Exercise Count}-\text{Rest Count}}{\text{Rest Count}}*100$$

## Data Availability

All data included in this manuscript are available from the corresponding author upon reasonable request.

## Key Points

- Exercise-mobilized donor lymphocytes (DLI-X) have enhanced activity against B-ALL in combination with Blinatumomab
- Enhanced anti-leukemic activity of DLI-X may be associated with upregulated T-cell costimulatory proteins CD137 and CD134
